# Changes in Pharmacokinetics and Pharmacodynamics of Losartan in Experimental Diseased Rats Treated with *Curcuma longa* and *Lepidium sativum*

**DOI:** 10.3390/ph16010033

**Published:** 2022-12-26

**Authors:** Abdul Ahad, Mohammad Raish, Ibrahim Abdelsalam Abdelrahman, Yousef A. Bin Jardan, Mohd Aftab Alam, Abdullah M. Al-Mohizea, Fahad I. Al-Jenoobi

**Affiliations:** Department of Pharmaceutics, College of Pharmacy, King Saud University, Riyadh 11451, Saudi Arabia

**Keywords:** garden cress, herb–drug interaction, hypertension, L-NAME, losartan, pharmacodynamic, pharmacokinetic, turmeric

## Abstract

The current study investigated “pharmacodynamics and pharmacokinetics interactions” of losartan with *Curcuma longa* (CUR) and *Lepidium sativum* (LS) in hypertensive rats. Hypertension was induced by oral administration of L-NAME (40 mg/kg) for two weeks. Oral administration of CUR or LS shows some substantial antihypertensive activity. The systolic blood pressure (SBP) of hypertensive rats was decreased by 7.04% and 8.78% 12 h after treatment with CUR and LS, respectively, as compared to rats treated with L-NAME alone. LS and CUR display the ability to potentiate the blood pressure-lowering effects of losartan in hypertensive rats. A greater decrease in SBP, by 11.66% and 13.74%, was observed in hypertensive rats treated with CUR + losartan and LS + losartan, respectively. Further, both the investigated herbs, CUR and LS, caused an increase in plasma concentrations of losartan in hypertensive rats. The AUC_0-t_, AUC_0-inf_ and AUMC_0-inf_ of losartan were increased by 1.25-fold, 1.28-fold and 1.09-fold in hypertensive rats treated with CUR + losartan. A significant (*p* < 0.05) increase in AUC_0-t_ (2.41-fold), AUC_0-inf_ (3.86-fold) and AUMC_0-inf_ (8.35-fold) of losartan was observed in hypertensive rats treated with LS + losartan. The present study affirms that interactions between CUR or LS with losartan alter both “pharmacokinetics and pharmacodynamics” of the drug. Concurrent administration of losartan with either CUR or LS would require dose adjustment and intermittent blood pressure monitoring for clinical use in hypertensive patients. Additional investigation is necessary to determine the importance of these interactions in humans and to elucidate the mechanisms of action behind these interactions.

## 1. Introduction

Medicinal herbs have been used for centuries as remedies in nearly all nations [1]. The consumption of medicinal plants to cure various ailments is common, particularly in developing countries [2,3,4]. Moreover, in Western countries, medicinal plant products are increasingly used, on the basis of self-selection, to either substitute for or supplement modern medicines [5,6,7]. Hence, the opportunity for herb–drug interactions is increasing as products are simultaneously administered [8,9,10]. Various interactions of antihypertensive drugs with concurrently administered herbs are reported [11,12,13]. Further, conventional herbs are being used increasingly by hypertensive patients and interactions of these herbs with antihypertensive medicines are now receiving notable attention [14,15,16,17,18].

Some studies have explored the use of herbal medicines for the management of hypertension and interactions with antihypertensive agents [8,19,20]. Several medicinal herbs cause alterations in blood pressure, and concurrent administration of herbs with modern antihypertensive drugs might modulate the effects of the drug [21,22]. Such interactions could result in hypertensive emergencies and other serious cardiovascular complications [17,23]. Thus, further studies are needed on the impacts of widely used herbs that could cause alteration in blood pressure and their “pharmacokinetics and pharmacodynamics interactions” with antihypertensive medications. Losartan is a frequently prescribed antihypertensive drug; a 50 mg tablet of losartan has a reported bioavailability of 32.6% [24]. Losartan works by inhibiting the angiotensin II receptor. It inhibits angiotensin II-induced physiological effects by binding competitively and selectively to the AT1 receptor. The drug also inhibits angiotensin II-induced vasoconstriction and aldosterone activity, thus lowering blood pressure [25,26]. It is likely that this low oral bioavailability is the result of inadequate absorption and inconsistent first-pass metabolism [27]. An estimated 14% of the losartan dose is metabolized into E 3174 after it is administered orally [28]. E 3174 is 10 to 40 times more potent than losartan itself; this metabolite is largely responsible for the pharmacological activity of losartan [29]. Losartan is metabolized predominantly by CYP3A4 and CYP2C9 [30,31,32]. Studies conducted in vitro with human liver microsomes have shown that at 10 μmol/L concentration, sulfaphenazole (inhibitor of CYP2C9) and ketoconazole (inhibitor of CYP3A4) inhibited the losartan metabolism to E 3174 by 81% and 12%, respectively [33,34]. Hence, modulation of the activity of these CYP enzymes by concurrent administration of herbs would alter the pharmacokinetics of losartan [35,36]. The present study examined two commonly used Asian herbal remedies viz. LS, (also known as garden cress) and CUR (also known as turmeric) for their impact on the “pharmacokinetics and pharmacodynamics” of losartan in hypertensive Wistar rats.

LS is an edible annual herb in the family Cruciferae, cropped in “India, Europe, and the United States” as well as “Arabian countries” [37,38,39].

A large portion of LS grows in West Asia and Egypt. Glucosinolates are the primary constituents of LS. In addition to volatile oils, the leaves and seeds of this plant are rich in minerals, amino acids and fatty acids [37,39]. There is 33–54% carbohydrates in seeds, 25% protein, 14–24% lipids and 8% crude fibre in seeds [40,41]. LS has been reported for treatment of several disorders [42,43,44]. Traditional herbal healers often suggest the herb for patients with high blood pressure, high blood glucose levels and renal disease [44,45,46]. Previously, Al-Jenoobi et al. investigated the effect of herbs, including LS, on the pharmacokinetics of theophylline in beagle dogs. The AUC_0-inf_ of theophylline increased (37%) when co-administered with LS [37].

In addition, simultaneous administration of LS changes sildenafil pharmacokinetics. Treatment of beagle dogs with LS leads to a substantial decrease in the C_max_ and AUC of sildenafil. Hence, LS administration might reduce the bioavailability of sildenafil and lessen its therapeutic effects [47]. Additionally, treatment of beagle dogs with LS caused a reduction in phenytoin clearance accompanied by an increase in the C_max_ and t_1/2_ of the drug [48]. A similar result was observed for the C_max_, and t_1/2_ of carbamazepine when co-administered with LS in rabbits [49].

“*Curcuma longa* L. commonly known as turmeric, (Zingiberaceae)” is known not only as a South-East Asian spice but also as a medicinal herb [50]. There are a number of countries that have used the rhizome of this plant as a safe remedy against a variety of ailments including sinusitis, coughs, wound healing, inflammation and skin problems [51,52,53,54]. The main components of turmeric are “bisdemethoxycurcumin (6%), demethoxycurcumin (17%) and curcumin (77%)” [50].

In previous study, concomitant oral administration of curcumin, the active constituent of CUR, and gliclazide in rats and rabbits showed that oral administration of curcumin potentiates the blood glucose-lowering action of gliclazide in normal and diabetic rats and in rabbits. No pharmacokinetic alteration in gliclazide was observed in animals pre-treated with curcumin [55]. In a different study, pre-treatment of rats with curcumin led to an increase in the C_max_ (3.5-fold) and AUC (1.7-fold) of losartan in rats [56]. Furthermore, the treatment of rats with curcumin and celiprolol produced an increase of 1.9 times in C_max_ and of 1.3 times in AUC [57].

Currently, few reports relate to “pharmacodynamic and pharmacokinetic interactions” of commonly used herbs, such as CUR and LS, with losartan. The present study addressed the hypothesis that pre-treatment of rats with CUR and LS will impact in vivo activity of losartan in L-NAME-induced hypertensive rats.

## 2. Materials and Methods

CUR was purchased from “Gul M. Memon spice factory, Jeddah, Saudi Arabia”. “L-NAME (*N*-nitro l-arginine methyl ester)” was procured from “Carbosynth limited, Berkshire, UK”. “Sortiva 50 mg (losartan potassium) was purchased from SPIMACO, Al-Qassim, Saudi Arabia”. LS was purchased from “Production of 7 Spices Trading establishment, Riyadh, Saudi Arabia”.

### 2.1. Induction of Hypertension in Rats

The L-NAME, at a dose of 40 mg/kg p.o., was administered to rats daily for two weeks for the induction of hypertension. Rats (250 ± 20 g) which showed systolic blood pressure (SBP) less than 150 mm Hg were excluded from the study [58,59]. The in vivo experiments were carried out on Wistar rats after the study was approved by the “Research Ethics Committee, King Saud University with approval number KSU-SE-18-27”.

### 2.2. Pharmacodynamics of Losartan in Hypertensive Rats Treated with CUR and LS

Rats were trained, once per day up to five days, to accommodate a restrainer so they remained calm during measurement of SBP [60,61,62]. The SBP was monitored with a “tail-cuff system (Visitech, BP-2000 series II, Apex, NC, USA)”.

Wistar rats were divided into three groups (Group A to C) with five animals in each group. Each group provided two sets of results: with and without losartan. In Group A, animals received L-NAME for two weeks. On day 14, SBP of each animal was monitored at 0, 1, 2, 4, 8 and 12 h (L-NAME alone). On day 15, animals were orally administered losartan (10 mg/kg, as single dose), and SBP was monitored 0, 1, 2, 4, 8 and 12 h (L-NAME + losartan) [63,64].

Similarly, Group B rats were treated with L-NAME + CUR (200 mg/kg/day) [65,66] for two weeks, and on day 14, SBP was monitored at 0, 1, 2, 4, 8 and 12 h (L-NAME + CUR). On day 15, animals were administered losartan (10 mg/kg, oral single dose) and SBP of each rat was monitored at 0, 1, 2, 4, 8 and 12 h (L-NAME + CUR + losartan). Group C rats were treated in the in the same manner as Group B animals, except that LS (300 mg/kg/day) was used instead of CUR [67,68,69].

### 2.3. Pharmacokinetics of Losartan in Hypertensive Rats Treated with CUR and LS

After a suitable washout period (three days) of Group A, B and C animals, losartan (10 mg/kg) was again administered as single oral dose, and blood samples were taken out from the animals at 0, 0.5, 1, 2, 4, 8 and 12 h. Plasma samples were analysed by UPLC-MS/MS [70]. During washout, Group A animals received L-NAME alone, and Group B and C animals were administered L-NAME + CUR and L-NAME + LS, respectively.

### 2.4. Statistical Analysis

Differences in means were analysed by one-way ANOVA followed by Tukey test using GraphPad Instant 3.06 (GraphPad Software, Inc, San Diego, CA, USA). * and # *p* < 0.05 were considered significant.

## 3. Results and Discussion

### 3.1. Changes in Losartan Pharmacodynamics in Hypertensive Rats Treated with CUR and LS

Medicinal herbs can induce or inhibit the drug-metabolizing enzymes, the modulating “pharmacokinetics and pharmacodynamics” drug substrates. CUR and LS can produce herb–drug interaction when administered concurrently with conventional drugs [66,71]. The current investigation aimed to measure the influence of CUR and LS on the blood pressure-lowering action and on the pharmacokinetic profile of losartan in L-NAME-induced hypertensive rats. Hypertension was induced with L-NAME, which is economical and widely used for this purpose in animal models. The study used hypertensive rats to mimic the human condition under which interactions might occur. In the current study, important alterations were noted in “pharmacodynamics and pharmacokinetics” of losartan with both the investigated herbs.

**Figure 1:**
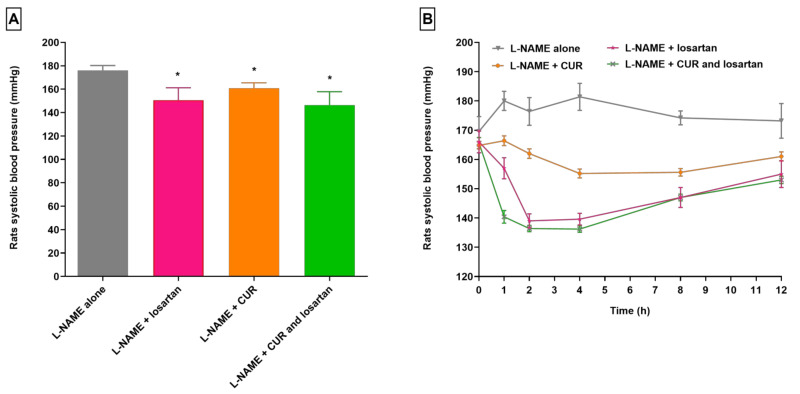
Changes in SBP in hypertensive rats receiving CUR. (**A**) Mean SBP after 12 h. (**B**) Time course of SBP (*n* = 5, mean ± SEM). CUR, *Curcuma longa*; L-NAME, “N-nitro l-arginine methyl ester”; SBP, systolic blood pressure. * *p* < 0.05 as compared to group treated with L-NAME alone.

**Figure 2:**
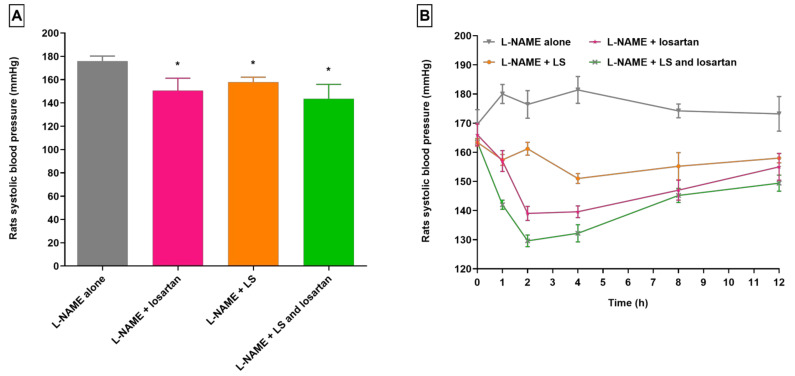
Changes in SBP in hypertensive rats receiving LS. (**A**) Mean SBP after 12 h. (**B**) Time course of SBP (*n* = 5, mean ± SEM). L-NAME, “N-nitro l-arginine methyl ester”; LS, *Lepidium sativum*; SBP, systolic blood pressure. * *p* < 0.05 as compared to group treated with L-NAME alone.

In normal rats, the SBP was 110.60 ± 3.32 mmHg (mean of 0 h to 12 h); an obvious rise in the SBP of rats (*p* < 0.05) was noted following the administration of L-NAME. Hypertension was effectively stimulated in all three groups of animals. Animals administered L-NAME alone exhibited an SBP of more than 150 mm Hg over the duration of the study. One hour after L-NAME administration, a 6.13% increment in SBP was observed, and SBP reached a maximum value of 181.40 mm Hg at 4 h (Figure 1 and Figure 2). A slight increase in SBP was noted with time, reaching 173.20 mg Hg at 12 h. Experimental animals that received L-NAME + losartan demonstrated a gradual decreased in SBP. Maximum SBP decreased from 166 mm Hg at 0 h to 139 mm Hg (*p* < 0.05) at 2 h after losartan administration. The SBP of rats increased steadily after 4 h following losartan administration; however, it remained as high as 155 mm Hg at 12 h. Treatment of experimental animals with L-NAME + CUR induced a gradual decline in the SBP of hypertensive rats through 12 h after CUR treatment. The maximum effect (*p* < 0.05) was seen at 4 h after CUR administration: SBP was measured at 164.80 mm Hg at 0 h, and at 155.20 mm Hg at 4 h, a 5.83% decrease. SBP decreased (at 4 h) by 14.44% compared to rats in the group treated with L-NAME alone (Figure 1). Four hours after CUR administration, a minor increase in SBP was observed, reaching 161 ± 1.581 mg Hg at 12 h. At 12 h, a 7.04% reduction in SBP in L-NAME + CUR-administered rats was noted when compared with the SBP reading at 12 h of rats administered L-NAME alone.

Animals administered L-NAME + CUR + losartan exhibited a sharp decline in SBP (*p* < 0.05, 136.20 ± 1.118 mm Hg) at 4 h after losartan administration. The decrease was 17.75% in comparison to the SBP of rats at 0 h, 165.60 ± 1.88 mm Hg. Beyond 2 h, the SBP of animals mildly raised, and then reached to 153.00 ± 1.22 mm Hg at 12 h. At 12 h, SBP was 11.66% less (*p* < 0.05) than SBP in animals in the group treated with L-NAME alone.

Likewise, animals treated with L-NAME + LS exhibited decreased SBP compared to rats in the L-NAME group. A significant decrease of 7.59% (*p* < 0.05) in the SBP of rats administered L-NAME + LS was observed at 4 h after administration of LS (Figure 2). Beyond 4 h, the SBP of rats progressively increased by 4.64% and reached 158.00 ± 1.64 mm Hg at 12 h. At 12 h, there was a decrease in SBP by 8.78% (*p* < 0.05) in rats treated with L-NAME + LS as compared with the 12 h reading of animals in the group treated with L-NAME alone.

In rats treated with L-NAME + LS + losartan, a drop of 20.69% in SBP was measured at 2 h after losartan administration. The SBP was noted to be 129.60 ± 2.01 mm Hg. From 2 h, the SBP of rats increased with time by 15.28%, and reached 149.40 ± 2.76 mm Hg after 12 h. This latter value was still 13.74% less (*p* < 0.05) than the SBP in rats in the group administered L-NAME alone. It was found that the everyday treatment of experimental rats with CUR and LS for two weeks enhances losartan effects on SBP. The findings of another study revealed that hypertensive animals treated with turmeric extract exhibited notable improvement in all the complications associated with hypertension investigated in this study. This improvement could be attributed to increased levels of nitric oxide and reduced arterial stiffness. The authors concluded that turmeric appears to play an active role in modulating vascular tone [72]. In another study, it was reported that the turmeric extract substantially lowered the blood pressure in Wistar rats. Animals treated with turmeric extracts (acute dose—5 g/kg) survived during the course of study. Further, the authors concluded that their study provides evidence that turmeric and its components exhibited a dose-dependent blood pressure-lowering effect, and that there is almost no toxicity associated with turmeric extract. Further, the primary active component of turmeric can mitigate oxidative stress by suppressing superoxide formation and enhancing glutathione. In addition, it elevated O_2_ generation and “endothelial nitric oxide synthase” levels in the arteries of hypertensive rats [73]. In another study, it was reported that the primary active component of turmeric (curcuma) suppresses angiotensin II receptor type 1 (AT1R) levels in A10 cells by modulating specificity protein 1/AT1R DNA binding, thereby decreasing AT1R-mediated vasoconstriction and, therefore, preventing the occurrence of blood pressure elevation in an angiotensin II-induced hypertensive experimental model [74]. In an antecedent investigation, Maghrani et al. showed that the treatment of “spontaneously hypertensive rats” with aqueous LS extract resulted in a substantial elevation in chloride, potassium and sodium excretion in the urine. The authors reported that regular intake of aqueous LS extract for a period of three weeks demonstrated diuretic as well as blood pressure-lowering effects [75]. Hence, based on the above observations, the substantial changes in the pharmacodynamic response of losartan with investigated herbs could be due to the improvement in the bioavailability of losartan, resulting in potentiation of the action of losartan in the presence of herbs.

### 3.2. Changes in Losartan Pharmacokinetics in Hypertensive Rats Treated with CUR and LS

**Figure 3:**
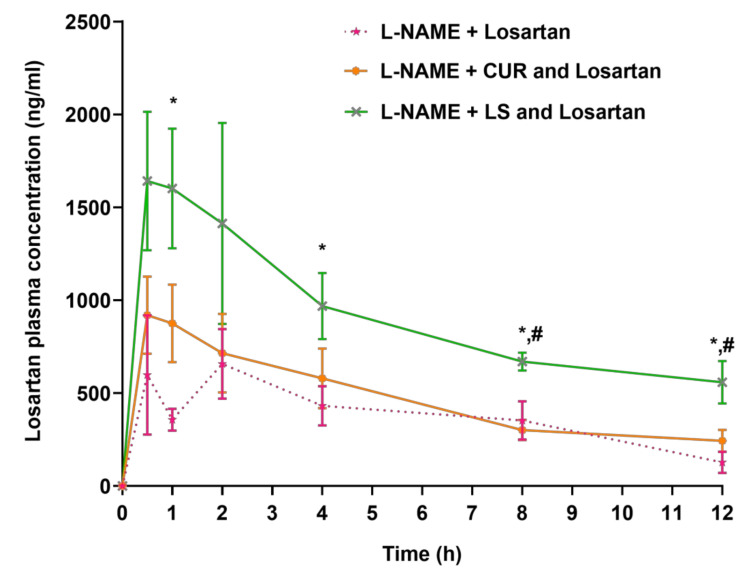
Pharmacokinetic profile of losartan in hypertensive rats treated with CUR and LS (*n* = 5, mean ± SEM). CUR, *Curcuma longa*; L-NAME, “N-nitro l-arginine methyl ester”; LS, *Lepidium sativum*. * *p* < 0.05 as compared to L-NAME + losartan, # *p* < 0.05 as compared to L-NAME + CUR+ losartan.

The plasma concentration versus time profiles of losartan with or without concomitant exposure to CUR and LS in L-NAME-induced hypertensive rats are provided in Figure 3.

**Figure 4:**
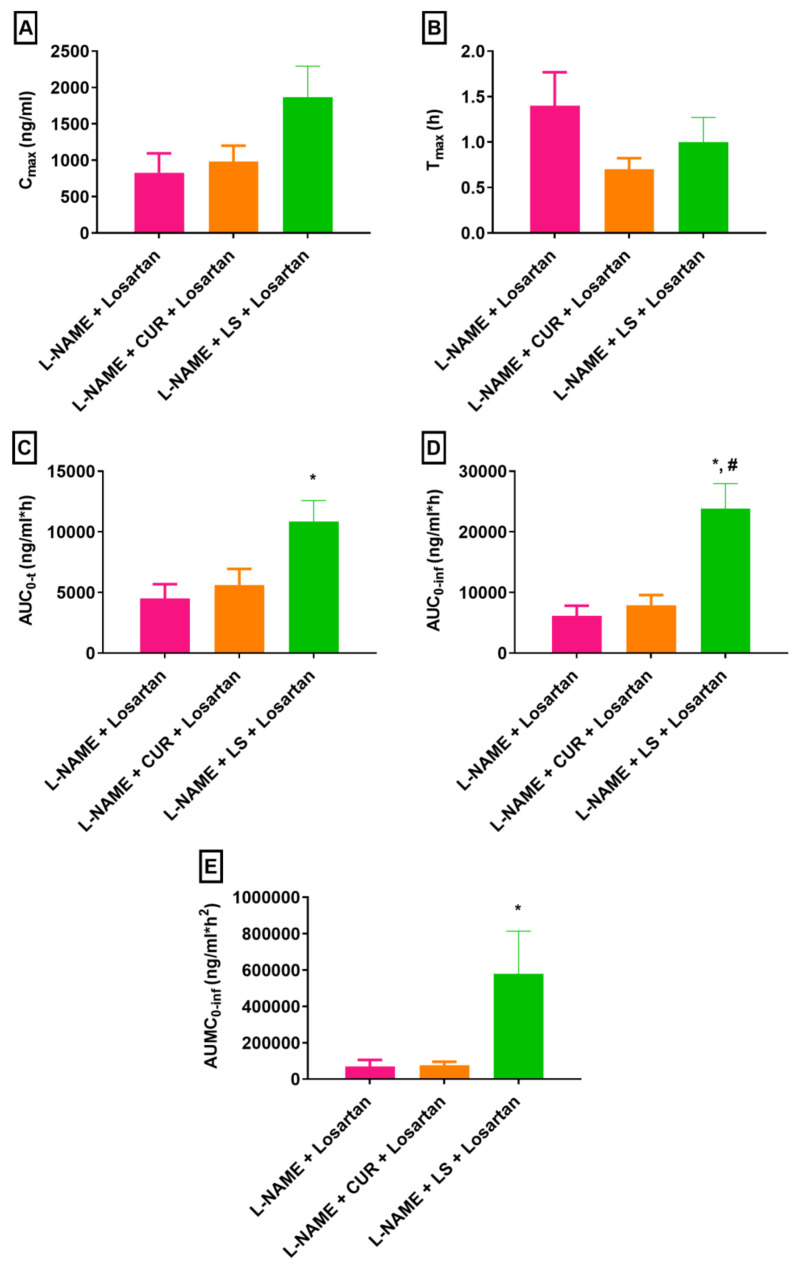
Pharmacokinetic parameters (**A**) C_max_, (**B**) T_max_, (**C**) AUC_0-t_, (**D**) AUC_0-inf_ and (**E**) AUMC_0-inf_ of losartan alone and in presence of CUR and LS following an oral administration in hypertensive rats (*n* = 5. mean ± SEM). L-NAME, “N-nitro l-arginine methyl ester”; CUR, *Curcuma longa*; LS, *Lepidium sativum*. * *p* < 0.05 as compared to L-NAME + losartan, # *p* < 0.05 as compared to L-NAME + CUR+ losartan.

A considerable increase in losartan C_max_ in animals treated with L-NAME + CUR + losartan and with L-NAME + LS + losartan was observed (Figure 4A). The C_max_ of losartan in animals treated with L-NAME + CUR + losartan and L-NAME + LS + losartan raised by 18.45% and 125.16%, respectively, in contrast to animals that received L-NAME + losartan, though these changes were not statistically significant (Figure 4A).

The T_max_ of losartan in rats treated with L-NAME + CUR + losartan and with L-NAME + LS + losartan were 0.70 ± 0.12 h and 1.00 ± 0.27 h, respectively (Figure 4B); while rats treated with L-NAME + losartan exhibited T_max_ of 1.40 ± 0.36 h (Figure 4B).

A 1.25-fold increase in the AUC_0-t_ of losartan plasma concentration was demonstrated in rats administered L-NAME + CUR + losartan in comparison to the control group animals (L-NAME + losartan); however, this change was not statistically significant (Figure 4C). On the other hand, the AUC_0-t_ of losartan in rats treated with L-NAME + LS + losartan was improved by 2.41-fold (*p* < 0.05) when compared with rats administered L-NAME + losartan (Figure 4C).

**Figure 5:**
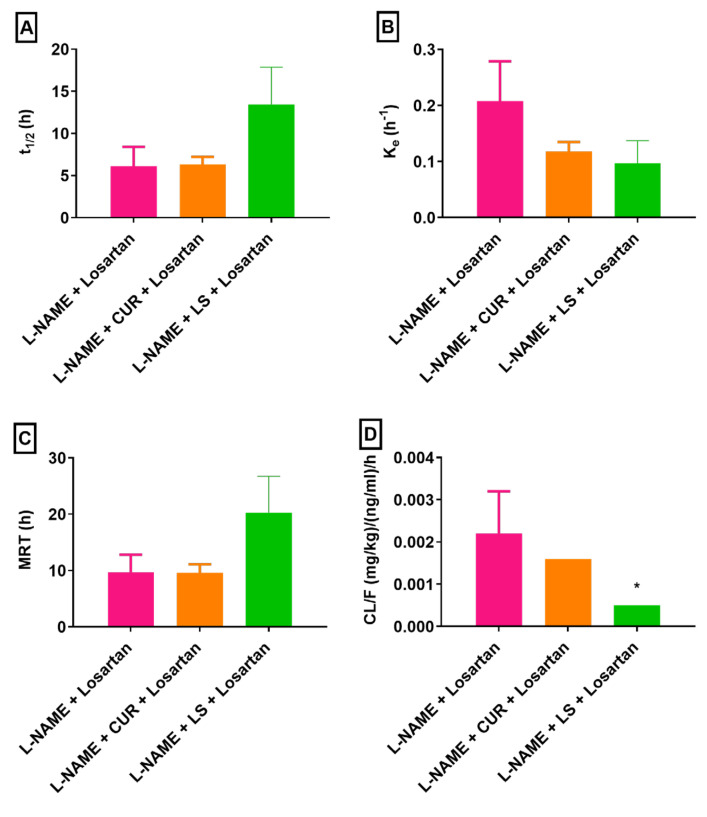
Pharmacokinetic parameters (**A**) t_1/2_, (**B**) K_e_, (**C**) MRT and (**D**) CL/F of losartan alone and in presence of CUR and LS following an oral administration in hypertensive rats (*n* = 5. mean ± SEM). L-NAME, “N-nitro l-arginine methyl ester”; CUR, *Curcuma longa*; LS, *Lepidium sativum*. * *p* < 0.05 as compared to L-NAME + losartan.

Further, a significant (*p* < 0.05) increase in the AUC_0-inf_ of losartan was noted in rats treated with L-NAME + LS + losartan, while the increase in the AUC_0-inf_ in rats treated with L-NAME + CUR + losartan was not statistically significant (Figure 4D). The AUMC_0-inf_ of losartan was also increased by 1.09-fold in rats that were administered L-NAME + CUR + losartan; meanwhile, a significant (*p* < 0.05) 8.35-fold increment in the AUMC_0-inf_ of losartan was noted in rats administered L-NAME + LS + losartan (Figure 4E). The t_1/2_ was extended by 1.04-fold and 2.21-fold in rats treated with L-NAME + CUR + losartan and with L-NAME + LS + losartan, respectively (Figure 5A). Inversely, the K_e_ of losartan was decreased by 43.22% and 53.16% (Figure 5B). The MRT of losartan was extended by 2.09-fold in animals treated with L-NAME + LS + losartan, while the MRT in groups treated with L-NAME + losartan and those treated with L-NAME + LS + losartan were comparable (Figure 5C). Meanwhile, the CL/F of losartan was decreased by 28.85% and 77.37% when animals were treated with L-NAME + CUR + losartan and with L-NAME + LS + losartan, respectively (Figure 5D). Both CUR and LS altered the pharmacokinetic parameters of losartan in rats; however, only the AUC_0-t_, AUC_0-inf_, AUMC_0-inf_ and MRT in rats treated with LS showed statistically significant changes. Other parameters did not show statistically significant changes with either herb, possibly due to high variation in pharmacokinetic data.

Previously, mostly studies have only demonstrated the blood pressure-lowering effect of investigated herbs. Only a few researches have been carried out related to the “pharmacodynamic and pharmacokinetic interactions” of commonly used herbs, such as CUR and LS, with losartan. Both herbs are very commonly ingested, particularly in developing countries, to cure various ailments, including hypertension. The current study indicates that both CUR and LS can alter both “pharmacodynamics and pharmacokinetics” of orally administered losartan in hypertensive rats. Oral administration of CUR or LS alone shows some significant antihypertensive activity. In addition, CUR and LS display the ability to potentiate the blood pressure-lowering effects of losartan in hypertensive rats. Further, both the investigated herbs, CUR and LS, caused an increase in plasma concentrations of losartan in hypertensive rats. A 1.25-fold and 2.41-fold increase in AUC_0-t_ of losartan was observed in hypertensive rats treated with L-NAME + CUR + losartan and with L-NAME + LS + losartan, as compared to the animals in a group treated with L-NAME + losartan. Hence, the efficacy of losartan could increase following the intentional or unintentional concomitant use of losartan and either herb. This could result in hypertensive emergencies and other serious cardiovascular complications. The dose of losartan might need to be adjusted when such use occurs. Despite this, additional studies are recommended in order to verify the clinical significance of these findings. We recommend additional in vivo research in animal models, studying other herbal plants that may affect blood pressure, in order to examine these herbs’ interactions with antihypertensive agents.

## 4. Conclusions

The present study was designed to examine “pharmacokinetic and pharmacodynamic interactions” of losartan concurrently administered with two common herbs, *Curcuma longa* and *Lepidium sativum,* in hypertensive rats. Hypertension was effectively induced in Wistar rats after oral administration of L-NAME. Administration of CUR and LS dropped the SBP of hypertensive rats by 7.04% and 8.78% at 12 h relative to hypertensive rats. A more pronounced decline in SBP, by 11.66% and 13.74%, was noted in hypertensive rats administered with CUR + losartan and LS + losartan, respectively. The AUC_0-t_ of losartan was improved by 1.25 times and 2.41 times in hypertensive rats treated with CUR + losartan and with LS + losartan, respectively. The study affirms that interaction of these herbs with losartan affects both the “pharmacokinetics and pharmacodynamics” of the drug. Hence, administration of either herb with losartan may require dose adjustment and monitoring of blood pressure at regular intervals. Additional investigation is needed to determine the probabilities of such herb–drug interactions in patients, and to define the mechanism of action.

## Data Availability

Data is contained within the article.
